# Experimental, Computational, and Machine Learning Methods for Prediction of Residual Stresses in Laser Additive Manufacturing: A Critical Review

**DOI:** 10.3390/ma17071498

**Published:** 2024-03-26

**Authors:** Sung-Heng Wu, Usman Tariq, Ranjit Joy, Todd Sparks, Aaron Flood, Frank Liou

**Affiliations:** 1 Department of Mechanical Engineering, Missouri University of Science and Technology, Rolla, MO 65409, USA; 2 Product Innovation and Engineering LLC, St. James, MO 65559, USA

**Keywords:** residual stresses, experimental measurement, computational method, machine learning

## Abstract

In recent decades, laser additive manufacturing has seen rapid development and has been applied to various fields, including the aerospace, automotive, and biomedical industries. However, the residual stresses that form during the manufacturing process can lead to defects in the printed parts, such as distortion and cracking. Therefore, accurately predicting residual stresses is crucial for preventing part failure and ensuring product quality. This critical review covers the fundamental aspects and formation mechanisms of residual stresses. It also extensively discusses the prediction of residual stresses utilizing experimental, computational, and machine learning methods. Finally, the review addresses the challenges and future directions in predicting residual stresses in laser additive manufacturing.

## 1. Introduction

Laser Additive Manufacturing (AM) is an emerging technology that employs focused laser beams to create complex geometries from a digital model, doing so layer by layer. This process offers unparalleled design freedom, rapid prototyping, and the ability to create internal structures [1]. Various techniques exist within additive manufacturing, each with unique applications and material compatibility. Laser Powder Bed Fusion (LPBF) is widely used in producing intricate parts and is ideal for materials like metal and plastic. Wire-Directed Energy Deposition (W-DED) involves the use of wire feedstock, offering benefits in terms of material usage and build rate. Powder-Directed Energy Deposition (P-DED) uses powder feedstock and is better suited for repair applications or multi-material layering [2].

**Figure 1:**
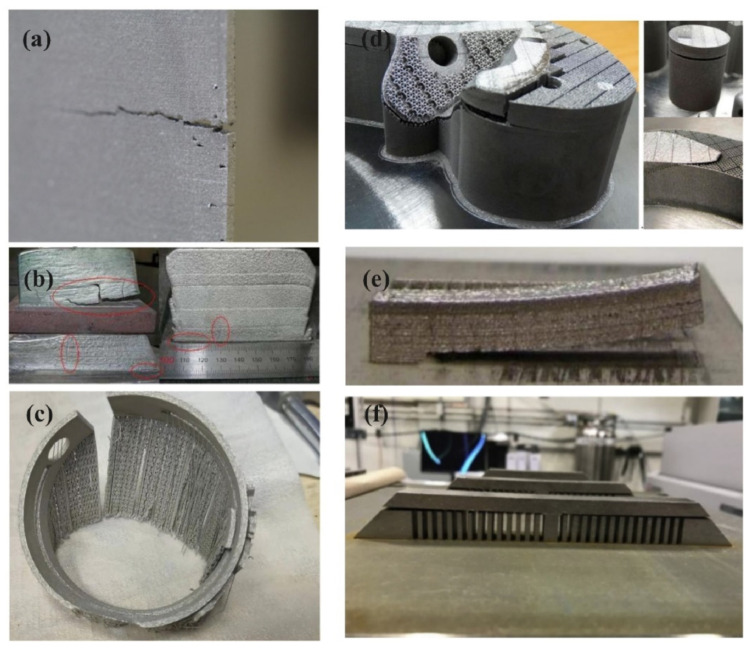
Effect of residual stresses in additive manufactured parts: (**a**–**d**) cracking, (**e**) delamination, and (**f**) distortion [17].

Laser additive manufacturing is also associated with various defects and disadvantages, such as lack of fusion, porosity, low surface finish, and dimensional tolerance. The formation of residual stresses during AM processes raises very critical issues, as it can lead to delamination, cracking, and early failure in AM parts [3,4]. Figure 1 shows various instances of part failure due to residual stress formation. Figure 1a–d shows cracking [5], whereas Figure 1e,f show delamination and distortion of AM parts, respectively [6,7]. Residual stresses in AM result from uneven cooling rates and thermal gradients during material deposition and solidification [8]. Residual stresses and distortion in AM can undermine part quality and integrity, posing significant challenges such as decreased fatigue strength, shrinking, and bending [9,10,11]. Potential applications of metal additive manufacturing include the manufacturing of complex free-form part designs and customization in aerospace [12,13,14], automotive [15], and biomedical applications [16]. Due to the critical nature of these applications, defects in AM parts could even lead to fatality. Residual stresses, in part, if not accounted for, can cause early failure during service, leading to detrimental effects on various stakeholders. Thus, these issues must be carefully researched and managed in the AM process.

**Figure 2:**
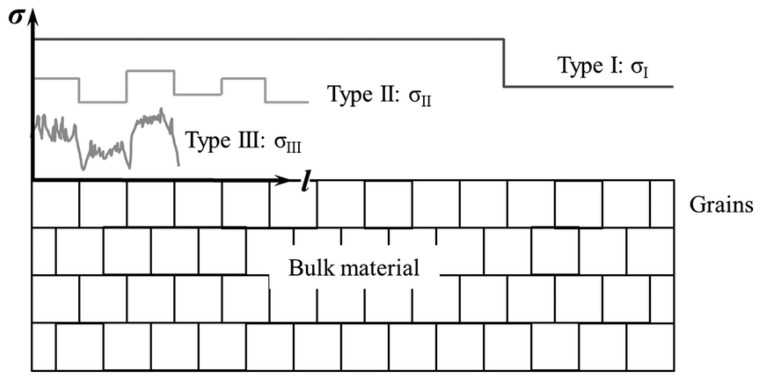
Three types of residual stresses [19].

Residual stresses naturally reach an equilibrium state, encompassing tensile residual stresses, which are often seen as detrimental, and compressive residual stresses, which are typically considered beneficial [18]. In AM, residual stresses manifest in the following three distinct types: Type I, macro-residual stresses, occur at a scale encompassing multiple grains; Type II, micro-residual stresses, develop within a single grain due to microstructural transformations; Type III, sub-micro-residual stresses, emerge within a few atomic units of the grain, influenced by factors like crystalline vacancies and dislocations [19], as shown in Figure 2.

Understanding the impact of residual stresses on part quality underscores the significance of both measuring and predicting residual stresses. This article provides an overview of various residual stress measurement methods, encompassing destructive, semi-destructive, and non-destructive techniques. Given the constraints of time and cost associated with experimental measurement, computational methods have gained popularity in recent years for predicting residual stresses prior to manufacturing. To enhance the versatility and control of computational approaches, analytical methods have also become increasingly prevalent. Moreover, statistical methods and machine learning have found applications in this research field, contributing to the efficiency of residual stress prediction.

The aim of this review is to provide readers with a comprehensive overview of current techniques and knowledge related to predicting residual stresses in additive manufacturing (AM) parts. The subsequent sections cover the fundamentals of residual stress and its formation, as well as the measurement and prediction of residual stresses using experimental, computational, analytical, and machine learning methods. Following this discussion, the authors outline several future research trends in this field.

## 2. Basics of Residual Stress and Its Formation

**Figure 3:**
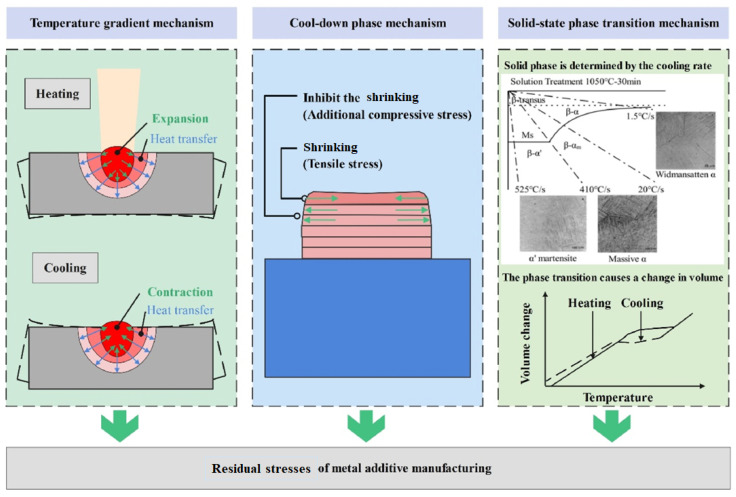
Different mechanisms of residual stress formation [17].

Residual stresses in laser additively manufactured parts can be categorized in three forms, as shown in Figure 3 [17,20]. The first phenomenon, known as the Thermal Gradient Mechanism (TGM), begins with localized heating and eventually creates a molten pool on the metal, while the remaining part of the metal plate maintains a normal temperature. This molten pool generates an expanding force due to its higher temperature, attempting to bend the plate outward. This localized bending often exceeds the elastic region of the part, causing plastic deformation [21]. The second stage, which is the cool-down phase (CDP) mechanism, involves the cooling of the molten pool. As the melted material tries to solidify, it contracts inward, causing the surrounding material to compress along with it, resulting in an inward distortion of the metallic plate.

The solid-state phase transition mechanism involves multiple phase transitions during the solidification process [22]. Depending on the cooling rate of certain materials, the ultimately solidified material may not have the same phase as it initially started with, resulting in the formation of residual stresses [23]. This review exclusively focuses on residual stress formation due to the first two mechanisms, namely TGM and CDP.

**Figure 4:**
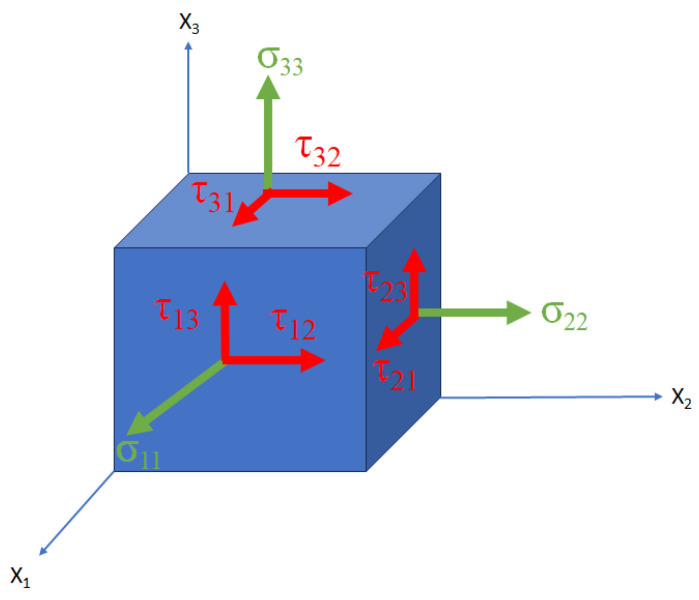
Distribution of Stress components in 3D.

Throughout the AM process, no external forces are applied; instead, all the stresses that arise stem from the laser heat source [24]. In simpler terms, residual stresses encompass the forces that persist within the deposit and substrate, even after all external operations are finished. These residual stresses can be the reason for the generation of strain and, consequently, deformation [25]. To determine whether these residual stresses surpass acceptable limits and could potentially result in distortion or part failure, various failure criteria must be satisfied. In many instances, stress components, maximum principal stress, and von Mises criteria are examined to assess part qualification [26]. Envisioning a single point within a 3D cube that is undergoing stresses, we can classify them into normal stresses and shear stresses, known as stress tensors. In Figure 4, certain tensor components are illustrated; these components play a pivotal role in comprehending each stress type on any plane, as expressed in Equations (1)–(3). The symbol σ is normal stress, and τ is shear stress, as shown in follow equations.

(1) $$\begin{array}{cc} \sigma_{33} & =\lim_{\Delta \to 0}\frac{\Delta F_{3}}{\Delta A} \end{array}$$

(2) $$\begin{array}{cc} \tau_{32} & =\lim_{\Delta \to 0}\frac{\Delta F_{2}}{\Delta A} \end{array}$$

(3) $$\begin{array}{cc} \tau_{31} & =\lim_{\Delta \to 0}\frac{\Delta F_{1}}{\Delta A} \end{array}$$

**Figure 5:**
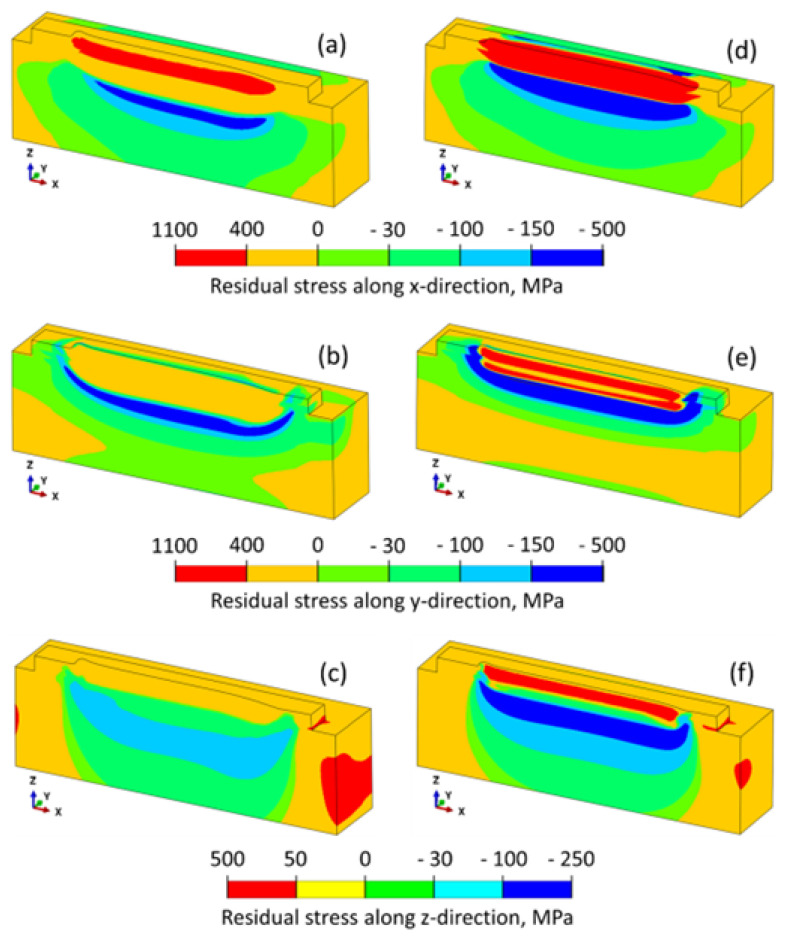
Stress distribution in x, y, and z directions during deposition of IN718 (**a**–**c**) and Ti64 (**d**–**f**) [27].

In Figure 5, materials IN718 and Ti64 use similar process parameters, i.e., a scan speed of 15 mm/s and laser power of 250 W. It was observed that for Ti64, the melt pool dimensions were larger as compared to IN718, creating more residual stresses in their respective directions [27].

**Figure 6:**
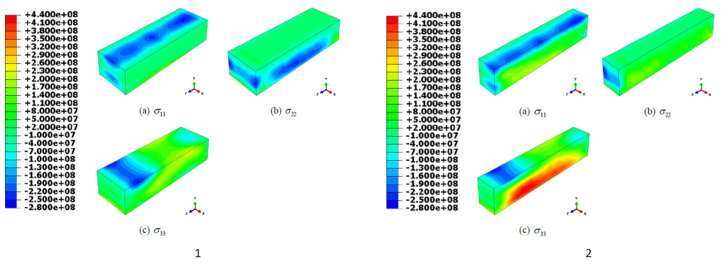
Directional stresses, in Pascal units, forecasted by Abaqus CAE in the x, y, and z planes on the deposit before sectioning in half (1) and after sectioning in half (2) [24].

Newkirk et al. conducted a numerical analysis by depositing 304 L using L-DED with three layers arranged in a zig-zag fashion, utilizing a laser power of 607 W and a scan speed of 250 mm/min. They also predicted the residual stresses [24]. Figure 6 illustrates the directional stresses forecasted by Abaqus CAE. It is noticeable that the majority of the tensile stresses occur in the longitudinal direction, possibly due to the cooling phase in the molten layers. During the solidification process, the remelted lower layers solidify, creating tensile stress due to restrictions imposed by the lower materials or previously solidified material.

Although the stress tensor explains the evolution of stresses in the respective directions, it is still essential to establish fail-safe criteria. Typically, for brittle materials, the maximum principal stress failure criterion is considered [28]. Three different variants of principal stress help understand the fracture location of the final part. The relationship between principal stress and the stress tensors in two dimensions is explained in Equation (4) [29].

(4) $$\begin{array}{c} \sigma_{1,2}=\frac{\sigma_{11}+\sigma_{22}}{2}\pm \sqrt{{\left(\frac{\sigma_{11}-\sigma_{22}}{2}\right)}^{2}+\tau_{12}^{2}} \end{array}$$

**Figure 7:**
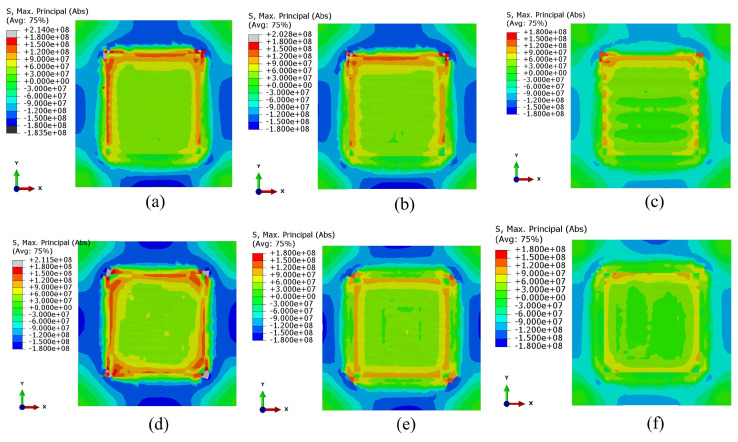
Distribution of maximum principal stresses, units in Pascal, using different scan strategies, (top view, unit: Pa): (**a**) zig-zag; (**b**) raster; (**c**) alternate line; (**d**) out–in spiral; (**e**) in–out spiral; (**f**) S. [26].

Li et al. [26] conducted a numerical analysis using a square substrate with a single layer to compare different scan strategies with the same process parameters. They concluded that a longer scan path would result in more residual stresses. Consequently, the spiral scan, with the longest track length, exhibited the highest principal stresses, while the S-scan type displayed the lowest principal stresses, as depicted in Figure 7 [26].

(5) $$\begin{array}{c} \tan2\theta_{P}=\frac{\tau_{12}}{(\sigma_{1}-\sigma_{2})/2} \end{array}$$

Having a failure criterion that addresses ductile materials is crucial because relying solely on the Maximum Principal Theory is inadequate for predicting the yield limit. The Maximum Distortion Energy Theory, also known as the von Mises criteria, asserts that yielding occurs when the combination of stress tensors surpasses the material’s yield point, as demonstrated in Equation (6) [29].

(6) $$\begin{array}{c} \sigma_{von}=\sqrt{\frac{1}{2}\left[{\left(\sigma_{11}-\sigma_{22}\right)}^{2}+{\left(\sigma_{22}-\sigma_{33}\right)}^{2}+{\left(\sigma_{33}-\sigma_{11}\right)}^{2}\right]+3\left[\left(\tau_{12}^{2}\right)+\left(\tau_{23}^{2}\right)+\left(\tau_{31}^{2}\right)\right]} \end{array}$$

**Figure 8:**
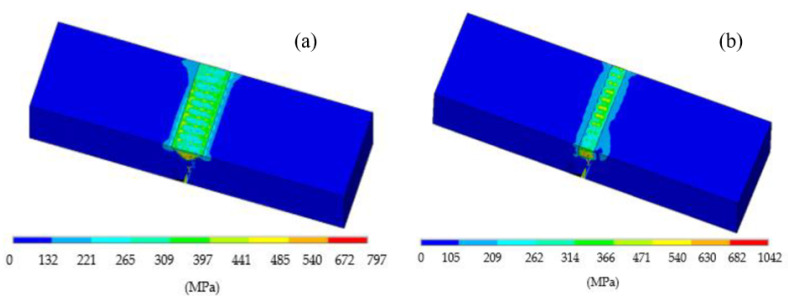
von Mises stress distributionwhile repairing using L-DEDin (**a**)V-shaped and (**b**) rectangular groove geometries [33].

Denlinger et al. conducted experiments on Ti64 and Inconel 625, utilizing identical process parameters but varying the interlayer dwell time from 0 to 40 s. They observed that increasing the dwell time led to an upsurge in von Mises stress for Ti64, while the opposite held true for Inconel 625 [30]. The variation in residual stresses between these two materials can be attributed to their structural distinctions [31]. Inconel maintains a face-centered cubic structure and does not undergo solid-state transformation, whereas Ti64 undergoes a two-phase (alpha–beta) allotropic solid-state transition [32]. In a related study, Lan Li et al. conducted experiments and numerical analyses to repair two geometries (V and rectangle-shaped), using powder DED technology with the same process parameters. It was noticed that more energy was transmitted in the rectangle shape, as it had a larger area to be deposited at the bottom, consequently exhibiting higher von Mises stresses, as shown in Figure 8. It was concluded that using a rectangle shape for repairs could lead to delamination as a result of exceeding the yield limit for Ti64 [33].

## 3. Experimental Method

Residual stresses in AM parts are often measured using various experimental methods [34]. Over the years, numerous researchers have worked on residual stresses measurement of traditionally manufactured parts [35]. Residual stress formation in welded joints has been well explored using various experimental methods [36,37]. These experimental methods are also utilized for RS measurement of AM parts. Most of these methods have been extensively studied and are well developed for practical applications [38]. This section provides a brief overview of the experiment-based RS measurement methods followed by a discussion of their capabilities and comparison for AM parts.

**Figure 9:**
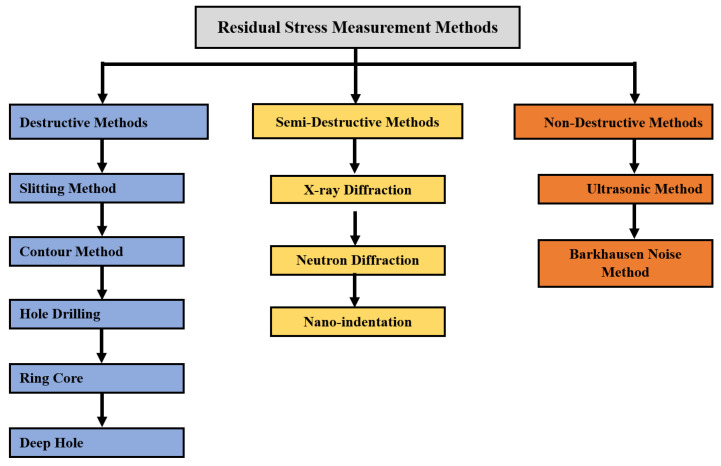
Experiment-based residual stress measurement methods.

As direct measurement of residual stresses in a manufactured part is not possible, measurement techniques often measure the strain or deformation of the final part and compare it with a non-stressed reference state [39,40]. Variation in the strain or material property can be correlated to the residual stress through established formulations such as Hooke’s law. These methods can be broadly classified into three categories, namely destructive, semi-destructive, and non-destructive methods. In destructive methods, a specimen completely loses its integrity as it is subjected to irreversible, macro-level alterations and cannot be used for its intended applications [41]. Some of the destructive methods include the contour method, hole drilling, the deep hole technique, etc. Non-destructive techniques such as the ultrasonic method and the Barkhausen noise method retain the integrity of the specimen after testing [42]. They analyze the variation of the material property in the stressed state to compute the stress values [43]. Semi-destructive methods can retain the integrity of the specimen to a certain extent but often lead to surface defects and micro-damage to the part, either during sample preparation or during the testing process [44]. X-ray diffraction and nanoindentation are examples of this category. Due to micro defects and damage, specimens are often discarded for critical applications. Figure 9 shows the classification of various experiment-based residual stress measurement methods, including destructive, semi-destructive, and non-destructive methods.

### 3.1. Destructive Methods

Destructive methods are often referred to as mechanical methods because they involve a stress relaxation procedure, and their fundamental principles of operation are similar. The presence of residual stresses in a part results in a distortion from the initial geometry and material removal from the part, which can lead to the relaxation of these stresses, followed by deformation to the initial geometry. Measurement of the magnitude and direction of this deformation can be correlated to the residual stress values of the part [41]. The most common examples of these destructive methods are detailed below.

#### 3.1.1. Slitting Method

The slitting method is used to determine the variation of residual stresses along the specimen thickness from the surface. As the name suggests, a thin slit or groove is made on the top surface, and the resulting strain relaxation is measured using strain gauges. The process is repeated with incremental increases in the depth of the slit [45]. Recorded strain data are used to map the stress profiles perpendicular to the cut surface using elasticity theory. The cutting method should not induce additional heating of the sample, which could release the stresses.

#### 3.1.2. Contour Method

The contour method also employs material removal for a stress relaxation procedure. In this method, a specimen is cut along the section where the stress has to be measured. Wire EDM is often used to ensure a that flat, ideal plane is obtained after cutting the contour. Relaxation and redistribution of the residual stress cause the contour to deform. The external reverse stress required to reinstate the plain state of the contour before cutting is calculated using simulation models. This external stress is considered equivalent to the residual stresses acting perpendicular to the cut surface [46]. The repeatability of this method for RS measurement is the same as or better than that of other methods [47].

#### 3.1.3. Hole-Drilling Method

Hole drilling is a very common and fast method used to obtain a 2D stress distribution. This method involves drilling a hole in the specimen surface and measuring the strain due to stress relaxation using strain gauges. It has excellent repeatability and is backed by the ASTM E837 standard [9]. The accuracy of the process depends on the quality of the drilling process.

#### 3.1.4. Ring Core Method

The ring core method can be thought of as a reversal of the hole-drilling method. In hole drilling, a hole is created, and the surrounding material on the outside is allowed to expand. However, in this method, a ring is machined, and the cylindrical specimen inside the hollow ring is allowed to deform, followed by strain measurements and RS calculations. This method offers larger surface strains but degrades the specimen significantly [9].

#### 3.1.5. Deep Hole Method

The deep hole method is a combination of the hole-drilling and ring core methods. The experimental procedure for this method begins with drilling a hole through the height of the sample. The diameter of this hole is accurately measured, followed by drilling a ring around it. This results in the deformation of the core material between the ring and the hole, changing the diameter of hole. This variation is used to calculate the stresses. All these methods are useful in hybrid manufacturing scenarios when there is a requirement to machine a hole in an additively manufactured part [45].

### 3.2. Semi-Destructive Methods

Semi-destructive methods employ either stress relaxation or material property comparison to determine the residual stresses. They do not destroy the specimen completely but often result in surface defects and sub-surface damage, which could potentially affect the performance of the part in critical applications such as the aerospace or biomedical fields.

#### 3.2.1. X-ray Diffraction

XRD is one of the most popular and widely used residual stress measurement methods. It is based on the variation of lattice spacing or inter-planar distance. Crystal lattices have characteristics of inter-planar distance during the unstressed state. An X-ray beam incident on the specimen surface is diffracted and captured by a detector at different reflective angles [48,49]. The lattice spacing of the specimen can be calculated using Bragg’s law. The change in the inter-planar spacing can be used to calculate the elastic strain, which can be utilized to calculate the magnitude and direction of residual stresses, provided the elastic properties of the material are known [50]. Different methods are utilized to extract the stress values from the diffraction readings, such as the sin2 method and the cos method [51,52].

Even though the operating mechanism of the XRD method does not cause any material destruction, the success of the method depends on the surface finish of the specimen. Sample preparation for XRD often involves surface polishing to obtain a micron-level surface finish, which can affect the residual stresses of the sample. Also, X-rays can penetrate to only few microns (<30 μm) [46]. Hence, to map the stress state of the material, layer-by-layer material removal is required, which destroys the specimen. High-energy synchrotron X-rays can be used to analyze Type III stress with nanometer-level penetration [53]. XRD combined with ion beam milling can be utilized to measure residual stresses [44,54]. However, the very high cost and scarcity of research facilities make it almost unavailable for most applications.

#### 3.2.2. Neutron Diffraction

Neutron diffraction is a method similar to XRD, employing the same principle for RS measurement. However, as the source irradiation is a neutron, it can penetrate into deeper sections, ranging from 25 mm for steel to 100 mm for aluminum. However, the availability of neutron diffraction equipment is very limited. Neutron diffraction can also employ the time-of-flight method to find the variation in lattice spacing for poly-crystalline materials. The time taken by the incident beam to be detected can be correlated to the lattice spacing. In this method, the incident angle and rotation angle are kept constant, but pulsed incident beams of different wavelengths are used [9].

#### 3.2.3. Nanoindentation

Nanoindentation utilizes the variation in localized hardness and elastic properties due to the presence of residual stresses. In this method, an indentation is made on the specimen surface at the nano scale. Tensile residual stresses allow for a larger contact area and deeper penetration, whereas compressive RS inversely affects the indentation. This method can be utilized to map the localized variation in AM samples but is limited to surface levels [46].

### 3.3. Non-Destructive Methods

Non-destructive methods maintain the integrity of the specimen or part, thus making it available for the intended use even after measurement. These methods compare a specific material property of the stressed part with a non-stressed reference state. Hence, no material removal is required for these methods.

#### 3.3.1. Ultrasonic Method

The ultrasonic method utilizes the acoustoelastic effect; the presence of applied or residual stresses in a solid varies the propagation characteristics of an acoustic wave. Compared to other non-destructive methods, the ultrasonic method can measure deeper stresses in the sample [55]. However, knowledge of acoustoelastic coefficients that define the linear relationship between stress and ultrasonic velocity is required prior to testing. Also, the change in ultrasonic velocities for each MPa of stress is very low (0.001%), which necessitates highly accurate measurement systems [53].

#### 3.3.2. Barkhausen Noise Method

The BNM is a non-destructive method applicable to ferromagnetic materials that can be magnetized. It is based on magneto-elastic interaction, where the elastic properties vary with the domain and magnetic property of the specimen [56]. Barkhausen noise, defined as the variation in electrical pulses induced in a coil due to a jump in the magnetic force field of the specimen when small-order magnetic domains are aligned parallel to the applied magnetic field, is analyzed for RS characterization [45]. Due to magneto-elastic interaction, for positive magnetic anisotropic materials such as iron, steels, and cobalt, compressive stresses tend to decrease Barkhausen noise intensity, whereas tensile stresses increases it [57].

BNM is also influenced by the microstructure of the specimen. Hence, initial calibration is highly significant in obtaining accurate results using this method [56]. This feature limits the the application of the BNM for AM parts, as the microstructure is highly non-homogeneous.

### 3.4. Comparison of Different Methods for AM Parts

**Figure 10:**
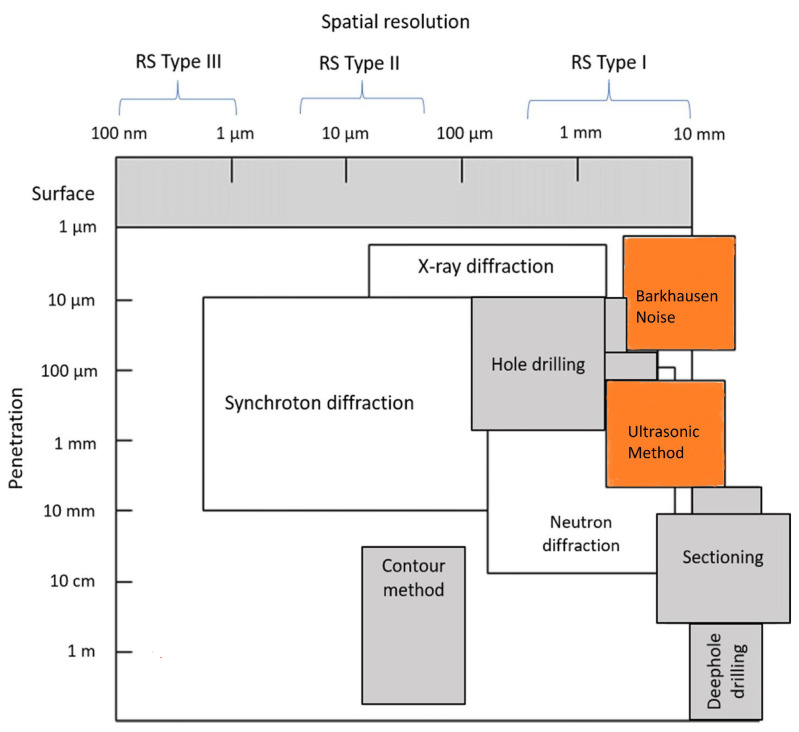
Capability of experiment-based RS measurement methods (gray–destructive, White–semi destructive, red–non destructive methods); adapted from [53].

Various methods used for measuring residual stresses were discussed previously. This section compares the discussed methods based on their advantages and limitations in measuring residual stresses in AM parts. Non-destructive methods are often preferred for AM parts, as they maintain the integrity of the part. However, RS measurement using non-destructive methods is associated with a lack of accuracy and difficulty in the experimental setup. Semi-destructive methods such as X-ray diffraction are widely utilized and are sometimes considered non-destructive. However, in reality, measurement using these methods depends on sample preparation and the dimensional limitations of the equipment [58]. Preparation of samples for XRD or neutron diffraction causes irreversible damage to the specimens, making these methods semi-destructive. Some of the widely used RS measurement methods are destructive or semi-destructive techniques. These are more mature technologies and can provide accurate, repeatable results. Additively manufactured parts lack repeatability compared to their conventional counterparts [59]. As a result, the standard testing procedure of randomly selecting a few specimens from a batch and extending the test results to the entire batch does not fit well [60]. Moreover, due to the intricate designs and high costs associated with producing AM parts, it is crucial to avoid any testing methods that could damage the parts’ structural integrity. This presents a decision-making scenario while choosing a method for measuring residual stresses, with benefits and disadvantages of different methods to be considered. Table 1 provides a summary of this comparison. Figure 10 provides an approximate map of the spatial resolution, penetration depth, and type of stress measured by various methods.

## 4. Computational Measurement Methods

### 4.1. Governing Equations of AM Processes

As discussed earlier, the formation of residual stresses in additive manufacturing (AM) parts is attributed to the laser heat source during both the heating and cooling processes. Therefore, this section can be divided into the following two parts: (1) thermal equations and (2) mechanical Equations.

#### 4.1.1. Thermal Model

The temperature distribution within a body, characterized by density (ρ) and specific heat (*C*), can be calculated by incorporating Fourier’s law of conduction, as shown in Equation (7) [24].

(7) $$\begin{array}{c} \rho C\frac{\partial T}{\partial t}=\frac{\partial }{\partial x}\left(k\frac{\partial T}{\partial x}\right)+\frac{\partial }{\partial y}\left(k\frac{\partial T}{\partial y}\right)+\frac{\partial }{\partial z}\left(k\frac{\partial T}{\partial z}\right)+Q \end{array}$$

In this equation, *T* represents temperature, *t* stands for time, *k* denotes thermal conductivity within the respective frame of reference, and *Q* represents internal heat generation. To solve this equation, proper initial and boundary conditions are necessary, as discussed below.

Various heat source models have been employed, and they can be essentially categorized into two types based on whether they use surface or body heat flux. Surface heat flux equations involve 2D heat flux that can be applied to the surface. A few examples include uniform heat sources, concentrated heat sources, and 2D Gaussian heat sources. The most commonly used body heat flux is Goldak’s double ellipsoidal heat source, as shown in Equation (8) [61].

(8) $$Q=\frac{6\sqrt{3}P\eta }{abc\sqrt{\pi }}exp\left(-\frac{3x^{2}}{a^{2}}-\frac{3y^{2}}{b^{2}}-\frac{3{\left(z+V_{s}t\right)}^{2}}{c^{2}}\right)$$

where *P* is power in Watts; η is the absorption co-efficient; a, b, and c are the width, depth, and length of the 3D Gaussian curve, respectively; and x, y, and z are the axes of the coordinate system, where the heat source moves in the z direction with respect to time (*t*) and velocity ($V_{s}$) [62].

During the additive manufacturing (AM) process, heat loss occurs through several methods, including convection and radiation. Among these, convection is a significant source of heat loss [63]. Convection mechanisms can be categorized as either free/natural or forced. Natural convection, which occurs when fluid or air movement is driven solely by buoyancy forces arising from temperature variations without external mechanical assistance, typically has values ranging from 5 to 15 W/m^2^C [64]. The measurement of free convection can be readily accomplished using either the lumped capacitance method or analytical techniques [65]. Forced convection happens when air/fluid moves due to external force. This phenomenon can be experienced by the melt pool due to shielding and the carrier gas of powder during deposition. Things get even more complex when deposition arms or fixtures are moving in different directions to perform 3D deposition; hence, this is difficult to measure and requires a wide range of experimentation [62]. In various literature reports, the peak of its value ranges from 40 to 120 W/m^2^C [66]. In some literature, this value was approximated as 18 W/m^2^C to obtain accurate results for thermal analysis [30]. For LPBF, it was observed that a forced convection value in the range of 5–20 W/m^2^C produced accurate models [67].

Heat loss due to radiation can be calculated using the Stephen–Boltzman law, as shown in Equation (9) [62].

(9) $$q_{rad}=\epsilon \sigma \left(T_{s}^{4}-T_{\infty }^{4}\right)$$

where ϵ is emissivity, σ is Stephen Boltzmann’s constant, and $T_{s}$ and $T_{\infty }$ represent the surface and room temperature, respectively. Sometimes, the above-mentioned loss of heat can be considered as a combined heat loss, as shown in Equations (10) and (11).

(10) $$\begin{array}{c} h=h_{free}+h_{forced}+h_{rad} \end{array}$$

(11) $$\begin{array}{c} q_{conv}=h\left(T_{s}-T_{\infty }\right) \end{array}$$

where $h_{free}$, $h_{forced}$ and $h_{radiation}$ are the coefficients of free, forced, and radiation heat transfer, respectively, and $q_{conv}$ is heat loss due to convection.

#### 4.1.2. Mechanical Model

The one-way or weakly coupled solution involves utilizing thermal data calculated in the preceding section to compute residual stresses in an additively manufactured part. For mechanical calculation, Equation (12) [66] serves as the equilibrium equation.

(12) ∇·σ+b=0

where σ is the stress tensor and **b** is body forces. A relationship is required to obtain information on stress and strain; hence, Equation (13) is used for that purpose.

(13) $$\sigma =C\epsilon_{e}$$

where $\epsilon_{e}$ is elastic strain and *C* is the fourth-order stiffness of the material [66]. In this process, residual stress forms due to strain induced by thermal or mechanical factors. When we expand these terms in the form of Equation (14), it appears as follows [24]:

(14) $$\epsilon_{ij}=\epsilon_{ij}^{E}+\epsilon_{ij}^{P}+\epsilon_{ij}^{T}+\epsilon_{ij}^{\Delta V}+\epsilon_{ij}^{Trp}$$

where $\epsilon_{ij}^{E}$ is strain due to elasticity, $\epsilon_{ij}^{P}$ is due to plasticity, $\epsilon_{ij}^{T}$ is due to thermal strain, $\epsilon_{ij}^{\Delta V}$ is due to volumetric change because of plastic transformation, and $\epsilon_{ij}^{Trp}$ is due to solid-state transition. $\epsilon_{ij}^{Trp}$ and $\epsilon_{ij}^{\Delta V}$ are due to solid-state phase transformation and are not discussed here so can be ignored for the purposes of this study.

### 4.2. Numerical Modeling Using FEA

To conduct numerical analysis, the governing equations are transformed into their weak form through the Galerkin method. The nodal values obtained from this weak formulation can then be employed to extract thermal data. The computation of thermal values first, followed by subsequent derivation of mechanical or stress values based on them is referred to as the one-way or decoupled method or the sequential method. This approach assumes that the mechanical model does not affect the thermal model [68]. One of the advantages of employing this method is that it reduces computational time compared to the coupled method. The partial differential equation of thermal analysis is parabolic to solve for thermal history, whereas for mechanical analysis it is of a quasi-static nature and is elliptical [62].

The addition of material can be mimicked using various methods, which include quiet activation, inactive activation, and hybrid activation.

The quiet-element method involves introducing elements representing metal deposition regions initially and assigning properties to minimize their impact. In heat transfer analyses, thermal conductivity (k) is reduced to limit conduction, and specific heat (Cp) is adjusted for energy transfer. This approach is easy to implement, maintaining a constant number of equations without the need for renumbering or solver initialization. However, inappropriate scaling factors can lead to errors from energy conduction or ill-conditioned Jacobians. In modeling additive manufacturing, where most of the domain consists of quiet elements, this method may result in lengthy computations [62].

The inactive-element method removes elements representing metal deposition regions from the analysis, focusing solely on nodal degrees of freedom for active elements. This approach avoids errors or ill-conditioning from scaling factors and results in smaller algebraic systems during Newton–Raphson linearization. However, it is less adaptable to general-purpose commercial codes, requiring repeated equation numbering and solver initialization when elements are activated and potentially introducing artificial energy when shared nodes have different initial temperatures [62].

The hybrid-element activation strategy was introduced as an enhancement over the quiet and inactive methods. Initially, elements are set as inactive, but they are gradually transitioned layer by layer to quiet, then to active, based on heat input. This approach minimizes equation numbering and solver initialization, leading to faster computations with comparable results [69].

### 4.3. Analytical Method

The analytical solution is the one that uses partial differential equations to solve for exact solutions. For example, a 1D governing equation that includes conduction and convection can be written as Equation (15) [62].

(15) $$-k\frac{dT}{dx}+h\left(T-T_{\infty }\right)=0$$

After solving the above differential equation for temperature, Equation (16) is obtained,

(16) $$T_{L}=\frac{\frac{k}{L}T_{0}+hT_{\infty }}{h+\frac{k}{L}}$$

where *L* is the length of the plate, $T_{L}$ is the temperature on that plate, and the remaining variables bear the same meanings as above. This method can not only be used to solve for temperature values of the 1D system but can also be used to verify the FE code to observe the error [62]. Ning et al. conducted analytical modeling to solve for thermal stresses, as shown in the equations below [70,71].

(17) $$\begin{array}{cc} \sigma_{xx}^{therm}\left(x,z\right) & =-\frac{\alpha E}{1-2v}\int_{0}^{\infty }\int_{-\infty }^{\infty }\left(G_{xh}\frac{\partial T}{\partial x}\left(x^{\prime },z^{\prime }\right)+G_{xv}\frac{\partial T}{\partial z}\left(x^{\prime },z^{\prime }\right)\right)dx^{\prime }dz^{\prime }\\ \; & +\frac{2z}{\pi }\int_{-\infty }^{\infty }\frac{p\left(t\right){\left(t-x\right)}^{2}}{{\left({\left(t-x\right)}^{2})+z^{2}\right)}^{2}}dt-\frac{\alpha ET(x,z)}{1-2v} \end{array}$$

(18) $$\begin{array}{cc} \sigma_{zz}^{therm}\left(x,z\right) & =-\frac{\alpha E}{1-2v}\int_{0}^{\infty }\int_{-\infty }^{\infty }\left(G_{zh}\frac{\partial T}{\partial x}\left(x^{\prime },z^{\prime }\right)+G_{zv}\frac{\partial T}{\partial z}\left(x^{\prime },z^{\prime }\right)\right)dx^{\prime }dz^{\prime }\\ \; & +\frac{2z^{3}}{\pi }\int_{-\infty }^{\infty }\frac{p(t)}{{\left({\left(t-x\right)}^{2})+z^{2}\right)}^{2}}dt-\frac{\alpha ET(x,z)}{1-2v} \end{array}$$

(19) $$\begin{array}{cc} \sigma_{xz}^{therm}\left(x,z\right) & =-\frac{\alpha E}{1-2v}\int_{0}^{\infty }\int_{-\infty }^{\infty }\left(G_{xzh}\frac{\partial T}{\partial x}\left(x^{\prime },z^{\prime }\right)+G_{xzv}\frac{\partial T}{\partial z}\left(x^{\prime },z^{\prime }\right)\right)dx^{\prime }dz^{\prime }\\ \; & +\frac{2z^{2}}{\pi }\int_{-\infty }^{\infty }\frac{p(t)(t-x)}{{\left({\left(t-x\right)}^{2})+z^{2}\right)}^{2}}dt \end{array}$$

(20) $$\begin{array}{cc} \sigma_{xz}^{therm}\left(x,z\right) & =v\left(\sigma_{xx}^{therm}+\sigma_{zz}^{therm}\right)-\alpha ET\left(x,z\right) \end{array}$$

(21) $$\begin{array}{cc} p(t) & =\frac{\alpha ET(x,z=0)}{1-2v} \end{array}$$

where the above-mentioned thermal stresses (σ) are calculated using Green’s function (*G*). Khan et al. [72] conducted analytical modeling and compared their results of residual stress prediction with the numerical method, as shown in Table 2.

Numerous studies have employed modeling techniques, utilizing both numerical and analytical methods, to calculate residual stresses in laser additive manufacturing, as presented in Table 3.

## 5. Machine Learning Method

The aforementioned reviews essentially show that the results of residual stresses calculated through computational methods are accurate and reliable. However, computational efficiency remains a significant challenge [86]. In order to facilitate the industrialization of additive manufacturing, it is crucial to create process simulation models capable of quickly forecasting the quality of parts. To address this issue, researchers have attempted to reduce the computation time by employing statistical methods and machine learning [87].

**Figure 11:**
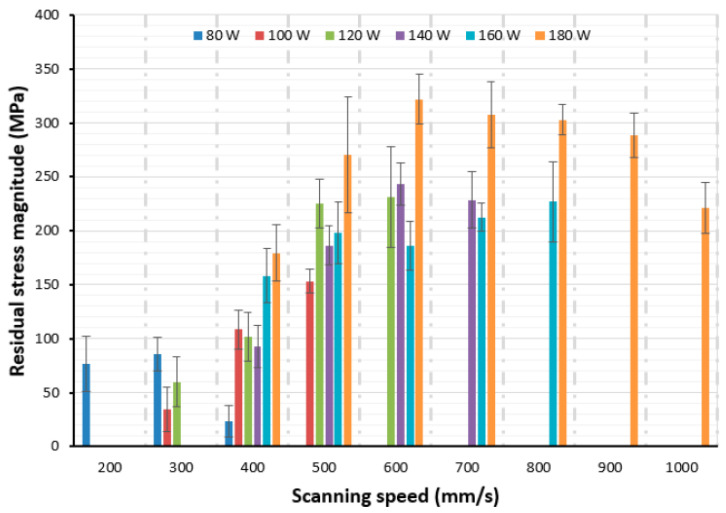
Effects of process parameters on residual stress [95].

Machine learning techniques are basically the data-driven methods for predicting the values that researchers expect. By using machine learning techniques to predict residual stresses and distortions, knowing what factors may affect the prediction is not mandatory but beneficial for collecting datasets and features for the training of machine learning models [88]. In the context of additive manufacturing, the intricate interplay of process parameters such as laser power, scanning speed, dwell time, building direction, and scanning strategy plays a pivotal role in influencing the formation of residual stresses within manufactured components [89,90]. Higher laser power, for instance, can increase the temperature gradient between melted and solidified layers, leading to significant thermal stresses that cool and contract differently across the part [91,92,93,94]. Conversely, scanning speed affects the heat input and cooling rates, where faster speeds may reduce the overall heat input and lead to uneven cooling rates, potentially increasing the likelihood of residual stress formation, as shown in Figure 11 [95,96]. Adjusting these parameters is crucial for managing thermal gradients and minimizing the internal stresses that can compromise the structural integrity and dimensional accuracy of 3D-printed parts.

Furthermore, dwell time, the delay between subsequent scans, and the building direction are critical factors affecting residual stress levels [30,58,97]. A longer dwell time allows for more heat dissipation into the surrounding material, potentially reducing thermal gradients but also increasing the risk of unwanted thermal effects if not carefully controlled [98,99]. The building direction influences the layer-by-layer construction of the part, with vertical or angled building directions affecting how heat accumulates and dissipates through the structure [100,101,102,103,104].

Strategic manipulation of these parameters can help control the cooling rates and thermal gradients, thus mitigating the formation of residual stresses [105]. Researchers have developed models and experimental studies to understand and predict the effects of these variables on residual stresses, emphasizing and strengthening the need for a machine learning technique to predict residual stresses and optimize additive manufacturing processes [106].

In this chapter, the article discusses various additive manufacturing processes, including welding, wire-arc additive manufacturing, laser powder-directed energy deposition, and laser powder bed fusion, to categorize and predict residual stresses using machine learning. Considering the correspondence between residual stresses and distortion, the prediction of distortion using machine learning techniques is also included in the following parts [107].

In the welding process, incorporating statistical methods to estimate residual stresses values, researchers have included parameters such as the depth of laser penetration and laser bead width into non-linear statistical regression analyses to predict residual stresses [107]. To further enhance the potential of obtaining residual stress predictions using machine learning, researchers have developed various algorithms with a wide array of input variables. In electron beam welding (EBW), input process parameters such as accelerating voltage, beam current, welding speed, and natural frequency have been considered [108]. Moreover, the heat source, cooling rate, and mechanical properties were considered in a series of research [109]. By implementing a variety of machine learning algorithms, M5 algorithm-based (M5P) model regression trees and multi-layer perceptions (MLPs) achieve better prediction performance [110]. Evolution fuzzy support vector regression (FSVG) can also achieve the accurate prediction of residual stresses [111]. Artificial neural networks (ANNs) and fuzzy neural networks (FNNs) have also been applied and compared to predict residual stresses, and FNNs were found to achieve better prediction accuracy [112].

**Figure 12:**
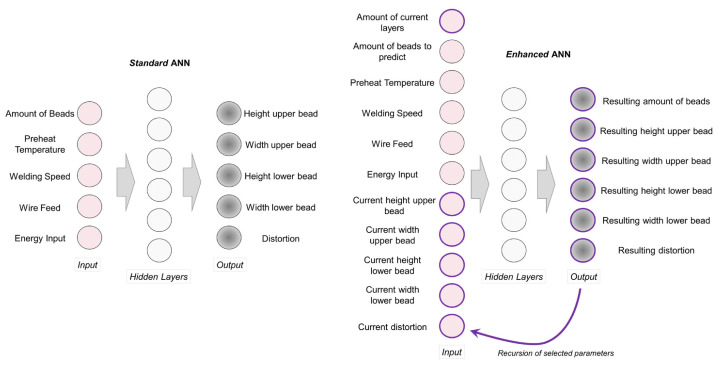
Input parameters of welding process [113].

**Figure 13:**
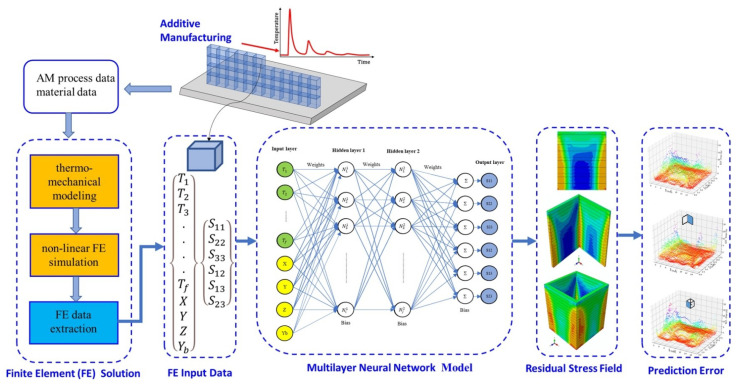
Integrating ANNs and FEA to predict RSS in LP-DED [116].

In wire-arc additive manufacturing (WAAM), standard and enhanced ANNs are implemented to train models to estimate distortion [113]. Process parameters such as the number of beads, preheating temperature, welding speed, wire feed, and energy are considered input parameters for the training model, as shown in Figure 12. Random forests (RFs) and ANN algorithms are applied to explore the hierarchy of influential variables, and the substrate preheating temperature is the most critical factor of residual stress [114]. Three levels of ANNs, namely the thermal history and field history of the deposition process, the cooling process, and the residual von Mises stress field, were developed to predict RSS, and the predicted time achieved the second level [115]. In laser powder-directed energy deposition (LP-DED), three classic geometries, namely a plane wall, L-shaped wall, and rectangular box, are included in finite element analysis with thermal–mechanical modeling to generate training datasets; then, an ANN is applied to build up a model to predict residual stresses [116], as shown in Figure 13. In addition to the ANN model, a convolution neural network is also applied to predict geometric deviation before deposition to avoid defects [117]. The gray-box model approach is also included in the shot-peening process. This gray-box-based model acts as the foundation for the machine learning technique by incorporating data from practical residual stress experiments, and the algorithm refines the initial model, steadily enhancing accuracy [118].

**Figure 14:**
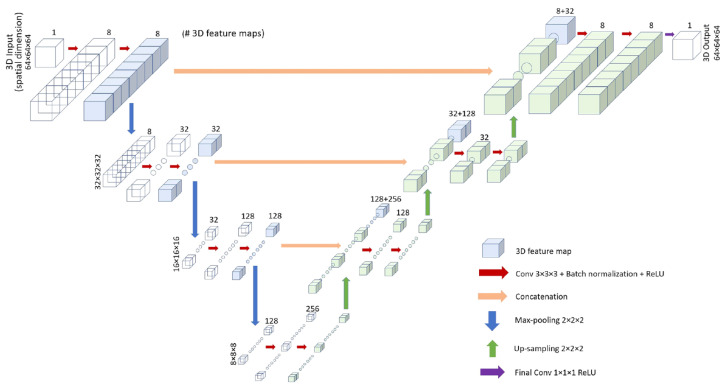
3D U-Net architecture [122].

In laser powder bed fusion (LPBF), distortion and residual stress are always accompanied by deposition and cause unqualified geometry. To mitigate those issues, finding the optimum value of process parameters such as laser power, scanning speed, and hatch spacing is critical [119]. Non-linear regression analysis is applied to obtain optimized process parameters to alleviate the residual stresses and distortion [120]. An ANN is applied to estimate the stress distribution on the cured layer from selective laser melting, achieving near-real-time results [121]. A convolution neural network (CNN) with a 3D U-Net architecture is applied to predict part-scale RSS, and three basic types of geometries are mutually combined by a full-order model (FOM) and included in training datasets [122], as shown in Figure 14. Considering that it is relatively challenging and time-consuming to collect data of residual stress, deep learning is also utilized to predict distortion during the LPBF process [123]. Additionally, real-time identification of layer-wise surface deformation of overhang geometries is presented by an imaged-based CNN algorithm [124]. A series of research has also utilized long-short term memory (LSTM) to predict real-time thermal history. The result was an acceleration of the efficiency of the finite element model in calculating residual stresses [125]. The researcher also compared the computational time between the machine learning and finite element models. One of the results showed that machine learning stress prediction required only about 0.47 s, which significantly less than the 5–10 h needed for finite element (FE) simulation [126]. Therefore, machine learning models have significant potential to integrate the existing data from simulations and experiments to predict residual stresses and deformation. The research studies that utilize machine learning methods to predict residual stresses and distortion are listed in Table 4.

## 6. Future Trends

Measurement of residual stresses of AM parts requires significant improvement in terms of accuracy, feasibility, and resolution. As discussed in the previous sections, these methods can be based on experiments, computation, or machine learning algorithms. While each of these categories has its own advantages, they lack in certain aspects.

Experiment-based methods involve measuring the strain or changes in material properties to calculate the residual stresses using elastic mechanics. However, these methods often require certain assumptions that limit the accuracy and applicability of the results. For example, the isotropic nature of the material and the absence of shear force are commonly adopted assumptions in experiment-based methods. As AM parts are largely anisotropic, experiment-based methods need to adopt modified stress–strain conversion. Also, damaging the integrity of AM parts during testing is highly undesirable, as batch-wise testing is not feasible for AM. Hence, pure non-destructive tests like the ultrasonic method or the Barkhausen noise method need to be adopted, and research on non-destructive methods needs to be explored further.

Numerical and analytical models are employed to predict the thermal history and distortion in laser additive manufacturing components. Numerical methods, while accurate, often demand substantial computational time. Researchers have begun implementing layer-based deposition techniques to alleviate this issue. However, this approach sacrifices some level of detail, reducing the accuracy of models. On the other hand, despite their limitation to two dimensions, analytical methods offer a trade-off between computational time and detail. Further exploration of analytical methods is warranted to incorporate all necessary dimensions for comprehensive modeling.

Prediction of residual stresses and distortion using machine learning methods involves a range of algorithms, as discussed previously, demonstrating their effectiveness in achieving accurate predictions. However, certain drawbacks and future research directions in this area are worth mentioning. First, concerning manufacturing processes, the majority of related studies have concentrated on welding, wire-arc additive manufacturing, and laser powder bed fusion. Consequently, it is desirable to expand research efforts into areas such as laser powder-directed energy deposition and electron beam welding.

Secondly, there is a need for finer and more reliable datasets. Most current research relies heavily on data from finite element analysis due to their abundance and the huge cost of collecting data in experiments. However, incorporating datasets from experiments can enhance the practicality of these datasets. Therefore, developing fast and accurate measurement methods is crucial and needed rapidly. Finally, in the realm of algorithms, exploring time-series algorithms like recurrent neural networks (RNNs) and long short-term memory (LSTM) networks is advisable because the distribution of residual stresses is essentially time-dependent. Moreover, given the complexity and the need for explainable AI models, the investigation of the use of physics-informed neural networks (PINNs) for prediction of residual stresses holds significant promise [128]. PINNs integrate physics and governing equations directly into the machine learning model. The loss function in PINNs includes partial differential equations (PDEs), boundary conditions, and initial conditions. Understanding the correspondence between thermal and mechanical models is crucial for the application of PINNs to predict residual stresses. Additionally, knowledge of material properties during additive manufacturing is also significant. Although some researchers have applied PINNs to analyze additive manufacturing processes for prediction of thermal distribution and melt pool characteristics, most research focuses on simple geometries, 2D dimensions, and the lack of residual stress analysis [129]. Therefore, there is potential to explore the prediction of residual stresses using PINNs [130].

Additionally, to enhance the versatility of the application, it is beneficial to consider a broader range of geometries in AI models. Improving the accuracy, efficiency, and compatibility of machine learning models is urgently required for further development in AM research, such as digital twins and digital factories, as it will be necessary to qualify AM parts for critical applications.

## 7. Conclusions

Residual stress evaluation in additively manufactured parts is critical, as residual stress leads to early failure and damages parts. The mechanism of residual stress formation in AM parts was discussed in this paper. Various methods used for residual stress evaluation were described, leading to the following conclusions:

- Experiment-based methods provide accurate results at the expense of the integrity of the part, which is highly undesirable. The development of easily accessible, non-destructive methods based on a matured theory that can measure different stress levels is required.
- Numerical modeling enables the prediction of residual stress and part distortion in three dimensions for various laser additive manufacturing processes. This versatility grants users the freedom to work with intricate geometries. However, it is associated with extended computational times and demands a high level of expertise to ensure model stability and prevent divergence.
- Machine learning and deep learning techniques have been employed to construct fast, predictive models for prediction of residual stresses in AM parts. They also provide the additional flexibility of in situ prediction of residual stresses. However, model accuracy is based on data developed by other methods, creating a dependency on experiment-based and computational methods.
- Future research directions were identified with respect to the potential development of a comprehensive method that incorporates experiment-based approaches, computational techniques, machine learning, and physics-informed neural networks. The interpretability provided by physics-informed algorithms can significantly enhance accuracy and reduce computation time, enabling better integration with experimental and computational models.

## Figures and Tables

**Table 1:** Comparison of RS measurement methods for AM parts; compiled from [45,46].

Method	Principle	Stress Type	Advantages	Limitations
Slitting Method	Strain release + elastic mechanics	Type I	Stress profile over entire specimen depth	Specimen destroyed; only stresses normal to cut surface
Contour Method	Strain release + Bueckner’s superposition principle	Type II	Wide range of materials; larger components; high-resolution maps	Destructive; immature theory; complex interpretation of data
Hole Drilling	Strain release + elastic mechanics	Type I	3 in-plane stresses; fast and easily available method; handheld equipment	Specimen destroyed; strain gauge affects accuracy
Ring Core	Strain release + elastic mechanics	Type I	Large depth measurement range; high accuracy	Significant damage to specimen; specialized equipment needed
Deep Hole Drilling	Strain release + elastic mechanics	Type I	Deep interior stress measurement; thick sections; wide range of materials	Specimen destroyed; interpretation of data; limited strain sensitivity
X-ray Diffraction	Lattice spacing variation + elastic mechanics	Type II	Matured technology; widely used method; high resolution	Works for crystalline materials with grains up to 100 microns; specimen texture controls accuracy; laboratory equipment
Slitting Method	Strain release + elastic mechanics	Type I	Stress profile over entire specimen depth	Specimen destroyed; only stresses normal to cut surface
Neutron Diffraction	Lattice spacing variation	Type I & Type II	Deep penetration and high resolution	Neutron source availability; lab-based system
Nanoindentation	Hardness variation, Hertz contact theory	Type II	High resolution for mapping of localized stress variation	Limited to surface stresses and thin films
Ultrasonic Method	Acoustoelastic effect	Type I	Independent of material, geometry, and texture; quick process; handheld equipment	Limited resolution; bulk measurements over large volume
Barkhausen Noise Method	Magnetoelastic interaction	Type I	Rapid process; no specimen contact; suitable for circular geometry	Only ferromagnetic materials; Microstructure affects measurement; MBN signal saturation limits range of measurable stresses

**Table 2:** Comparison of computational time for prediction of residual stresses during LPBF deposition using numerical and analytical methods [72].

Dimensions (mm^3^)	Simulation Details			Single-Core Run Time (h)	
	Computed Layers	Nodes	Elements	Numerical	Analytical
35 × 15 × 0.15	1	111,908	63,820	8.4 [73]	0.0003
50 × 5 × 50	100	495,504	494,010	29.4 [74]	0.0833
20 × 10 × 10	200	344,750	329,250	9280 [75]	0.015

**Table 3:** Numerical and analytical models used in laser additive manufacturing for the prediction of residual stresses for Selective Laser Melting (SLM), Powder Bed Fusion (PBF) and Laser Powder Directed Energy Deposition (LPDED).

Technology	Beam Dia (mm)	Scale (mm^3^)	Method	Elements	Computer	Compute Time (h)	Ref.
SLM	0.4	6 × 6 × 0.09	Numerical/Abaqus	20,800	Xeon E5	72 (thermal) + 20 (mechanical)	[76]
SLM	0.08	0.5 × 0.5 × 0.2	Numerical/ANSYS (APDL)	*	*	*	[77]
SLM	2	20 × 20 × 4	Numerical/ANSYS	200	*	*	[78]
SLM	0.07	1.19 × 0.315 × 0.2175	Numerical/ANSYS (APDL)	*	*	*	[79]
SLM	0.05	1.92 × 0.48 × 0.08	Numerical/In-house developed	*	*	*	[80]
SLM	0.07	3 × 3 × 0.05 3 × 3 × 0.250 3 × 3 × 1.250	Analytical	NA	*	*	[81]
PBF	0.15	40 × 5 × 2	Analytical/Matlab	NA	2.8 GHz	7.26 s	[70]
PBF	0.054	10 × 5 × 5	Analytical	NA	*	*	[82]
PBF	*	20 × 10 × 3	Analytical	NA	4 cores	45 s	[82]
LPDED	0.74	12 × 5 × 12	Numerical/Abaqus	343,728	8 cores 2.1 GHz	216	[83]
SLM	0.4	6 × 6 × 0.09	Numerical/Abaqus	20,800	Xeon E5	72 (thermal) + 20 (mechanical)	[76]
SLM	0.08	0.5 × 0.5 × 0.2	Numerical/ANSYS (APDL)	*	*	*	[77]
LPDED	3	20 × 80 × 4	Numerical/COMET	19,040	*	*	[84]
LPDED	5	100 × 5 × 3	Numerical/Abaqus	*	*	*	[85]

* Unspecified in the original source.

**Table 4:** Predicting residual stresses and distortion using machine learning.

Process	Material	Data Source	Prediction	Algorithm	Ref.
Welding	Al Alloy	Experiments	Distortion	Linear regression	[107]
EBW	SS304	Experiments	RSS	M5P, SVR	[110]
Welding	Mild Steel	FEM	RSS	FSVG	[111]
Welding	Stainless Steel	Experiments	RSS	ANN, FNN	[112]
WAAM	Iron	Experiments	Distortion	Enhanced ANN	[113]
WAAM	SS316, IN718	Experiments FEM	RSS	RF, ANN	[114]
WAAM	ER308L	FEM	RSS	Three-level ANNs	[115]
LPBF	AlSi10Mg	FEM	Distortion	Multiple regression	[120]
LPBF	Unspecified	CAD Drawings	RSS	3D U-Net CNN	[127]
LPBF	Ti-6Al-4V	Experiments	Distortion	CNN	[123]
LPBF	AlSi10MG	Experiments	Distortion	CNN	[124]
LP-DED	SS304L	FEM	RSS	ANN	[116]

## Data Availability

No new data were created.

## References

[1] Liou F.W. (2007). Rapid Prototyping and Engineering Applications: A Toolbox for Prototype Development.

[2] Wong K.V., Hernandez A. (2012). A Review of Additive Manufacturing. ISRN Mech. Eng..

[3] Yadroitsev I., Yadroitsava I. (2015). Evaluation of residual stress in stainless steel 316L and Ti6Al4V samples produced by selective laser melting. Virtual Phys. Prototyp..

[4] Lai Y.B., Liu W.J., Zhao J.B., Zhao Y.H., Wang F.Y., Han W.C. (2013). Experimental Study on Residual Stress in Titanium Alloy Laser Additive Manufacturing. Appl. Mech. Mater..

[5] Parry L., Ashcroft I.A., Wildman R.D. (2016). Understanding the effect of laser scan strategy on residual stress in selective laser melting through thermo-mechanical simulation. Addit. Manuf..

[6] Levkulich N.C. (2017). An Experimental Investigation of Residual Stress Development during Selective Laser Melting of Ti-6Al-4V. Master’s Thesis.

[7] Chen Q., Liang X., Hayduke D., Liu J., Cheng L., Oskin J., Whitmore R., To A.C. (2019). An inherent strain based multiscale modeling framework for simulating part-scale residual deformation for direct metal laser sintering. Addit. Manuf..

[8] Li C., Liu Z.Y., Fang X.Y., Guo Y.B. (2018). Residual Stress in Metal Additive Manufacturing. Procedia CIRP.

[9] Carpenter K., Tabei A. (2020). On residual stress development, prevention, and compensation in metal additive manufacturing. Materials.

[10] Liao D., Zhu S.P., Keshtegar B., Qian G., Wang Q. (2020). Probabilistic framework for fatigue life assessment of notched components under size effects. Int. J. Mech. Sci..

[11] Wang Y.X., Hung C.H., Pommerenke H., Wu S.H., Liu T.Y. (2024). Fabrication of crack-free aluminum alloy 6061 parts using laser foil printing process. Rapid Prototyp. J..

[12] Wilson J.M., Piya C., Shin Y.C., Zhao F., Ramani K. (2014). Remanufacturing of turbine blades by laser direct deposition with its energy and environmental impact analysis. J. Clean. Prod..

[13] Singamneni S., LV Y., Hewitt A., Chalk R., Thomas W., Jordison D. (2019). Additive Manufacturing for the Aircraft Industry: A Review. J. Aeronaut. Aerosp. Eng..

[14] Valiev S., Okafor A.C., Prakash Hungund A., Huang J. (2024). Identification of damage and corrosion effect in aging aircraft data transmission lines. Eng. Fail. Anal..

[15] Wischeropp T.M., Hoch H., Beckmann F., Emmelmann C. (2019). Opportunities for Braking Technology Due to Additive Manufacturing through the Example of a Bugatti Brake Caliper. Proceedings of the XXXVII International μ-Symposium 2018 Brake Conference 2018.

[16] Harrysson O.L., Marcellin-Little D.J., Horn T.J. (2015). Applications of Metal Additive Manufacturing in Veterinary Orthopedic Surgery. JOM.

[17] Chen S.g., Gao H.j., Zhang Y.d., Wu Q., Gao Z.h., Zhou X. (2022). Review on residual stresses in metal additive manufacturing: Formation mechanisms, parameter dependencies, prediction and control approaches. J. Mater. Res. Technol..

[18] Bartlett J.L., Li X. (2019). An overview of residual stresses in metal powder bed fusion. Addit. Manuf..

[19] Liu D., Flewitt P.E. (2014). Raman Measurements of Stress in Films and Coatings.

[20] Mercelis P., Kruth J.P. (2006). Residual stresses in selective laser sintering and selective laser melting. Rapid Prototyp. J..

[21] Luo Y., Liu X., Chen F., Zhang H., Xiao X. (2023). Numerical simulation on crack–inclusion interaction for rib-to-deck welded joints in orthotropic steel deck. Metals.

[22] Shen Z., Dong R., Li J., Su Y., Long X. (2024). Determination of gradient residual stress for elastoplastic materials by nanoindentation. J. Manuf. Processes.

[23] Chen F., Zhang H., Li Z., Luo Y., Xiao X., Liu Y. (2024). Residual stresses effects on fatigue crack growth behavior of rib-to-deck double-sided welded joints in orthotropic steel decks. Adv. Struct. Eng..

[24] Newkirk J.W. (2014). Multi-Layer Laser Metal Deposition Process. Master’s Thesis.

[25] Dupont J.N. (2018). Fundamentals of Weld Solidification. Weld. Fundam. Processes.

[26] Sun L., Ren X., He J., Zhang Z. (2021). Numerical investigation of a novel pattern for reducing residual stress in metal additive manufacturing. Mater. Sci. Technol..

[27] Mukherjee T., Zhang W., DebRoy T. (2017). An improved prediction of residual stresses and distortion in additive manufacturing. Comput. Mater. Sci..

[28] Chen Y., Sun S., Zhang T., Zhou X., Li S. (2020). Effects of post-weld heat treatment on the microstructure and mechanical properties of laser-welded NiTi/304SS joint with Ni filler. Mater. Sci. Eng. A.

[29] Todo M. (2015). Mechanics of Materials.

[30] Denlinger E.R., Heigel J.C., Michaleris P., Palmer T.A. (2015). Effect of inter-layer dwell time on distortion and residual stress in additive manufacturing of titanium and nickel alloys. J. Mater. Process. Technol..

[31] Yang Z., Tang B., Qiu Y., Wu J., Wei W., Huang X., Luo X., Wu G. (2023). Measurement of transient temperature using laser-induced breakdown spectroscopy (LIBS) with the surface temperature effect. J. Anal. At. Spectrom..

[32] Jiang X., Bao S., Zhang L., Zhang X., Jiao L., Qi H., Wang F. (2023). Effect of Zr on microstructure and properties of TC4 alloy fabricated by laser additive manufacturing. J. Mater. Res. Technol..

[33] Li L., Zhang X., Liou F. (2021). Experimental and numerical investigation in directed energy deposition for component repair. Materials.

[34] Withers P.J., Bhadeshia H.K. (2001). Residual stress part 1–Measurement techniques. Mater. Sci. Technol..

[35] Huang X., Liu Z., Xie H. (2013). Recent progress in residual stress measurement techniques. Acta Mech. Solida Sin..

[36] Anawa E.M., Olabi A.G. (2008). Control of welding residual stress for dissimilar laser welded materials. J. Mater. Process. Technol..

[37] Bahadur A., Kumar B.R., Kumar A.S., Sarkar G.G., Rao J.S. (2004). Development and comparison of residual stress measurement on welds by various methods. Mater. Sci. Technol..

[38] Tabatabaeian A., Ghasemi A.R., Shokrieh M.M., Marzbanrad B., Baraheni M., Fotouhi M. (2022). Residual Stress in Engineering Materials: A Review. Adv. Eng. Mater..

[39] Guo Y., Wang L., Zhang Z., Cao J., Xia X., Liu Y. (2024). Integrated modeling for retired mechanical product genes in remanufacturing: A knowledge graph-based approach. Adv. Eng. Inform..

[40] Li X.K., Zhu S.P., Liao D., Correia J.A., Berto F., Wang Q. (2022). Probabilistic fatigue modelling of metallic materials under notch and size effect using the weakest link theory. Int. J. Fatigue.

[41] Schajer G.S. (2010). Relaxation Methods for Measuring Residual Stresses: Techniques and Opportunities. Exp. Mech..

[42] Dive V., Lakade S. (2021). Recent Research Progress on Residual Stress Measurement Using Non-Destructive Testing. Mater. Today Proc..

[43] Zhu Q., Chen J., Gou G., Chen H., Li P. (2017). Ameliorated longitudinal critically refracted—Attenuation velocity method for welding residual stress measurement. J. Mater. Process. Technol..

[44] Burns E., Newkirk J., Castle J., Creamer J. (2019). Micro-slotting Residual Stress Measurement Technique for Understanding Fatigue Performance of Open-Hole Ti-6Al-4V Samples. J. Mater. Eng. Perform..

[45] Rossini N.S., Dassisti M., Benyounis K.Y., Olabi A.G. (2012). Methods of measuring residual stresses in components. Mater. Des..

[46] Guo J., Fu H., Pan B., Kang R. (2021). Recent progress of residual stress measurement methods: A review. Chin. J. Aeronaut..

[47] Hill M.R., Olson M.D. (2014). Repeatability of the Contour Method for Residual Stress Measurement. Exp. Mech..

[48] Song J., Chen Y., Hao X., Wang M., Ma Y., Xie J. (2024). Microstructure and mechanical properties of novel Ni–Cr–Co-based superalloy GTAW joints. J. Mater. Res. Technol..

[49] Hua L., Liu Y., Qian D., Xie L., Wang F., Wu M. (2022). Mechanism of void healing in cold rolled aeroengine M50 bearing steel under electroshocking treatment: A combined experimental and simulation study. Mater. Charact..

[50] Schajer G.S., Prime M.B., Withers P.J. (2022). Why Is It So Challenging to Measure Residual Stresses ?. Exp. Mech..

[51] Tanaka K. (2019). The cos*α* method for X-ray residual stress measurement using two-dimensional detector. Mech. Eng. Rev..

[52] Sarmast A., Schubnell J., Preußner J., Hinterstein M., Carl E. (2023). Residual stress analysis in industrial parts: A comprehensive comparison of XRD methods. J. Mater. Sci..

[53] Acevedo R., Sedlak P., Kolman R., Fredel M. (2020). Residual stress analysis of additive manufacturing of metallic parts using ultrasonic waves: State of the art review. J. Mater. Res. Technol..

[54] Burns E., Newkirk J., Castle J. (2018). Micro-slotting technique for reliable measurement of sub-surface residual stress in Ti-6Al-4V. J. Strain Anal. Eng. Des..

[55] Castellano A., Fraddosio A., Marzano S., Daniele Piccioni M. (2017). Some advancements in the ultrasonic evaluation of initial stress states by the analysis of the acousto-elastic effect. Procedia Eng..

[56] Gur C.H. (2018). Review of residual stress measurement by magnetic Barkhausen noise technique. Mater. Perform. Charact..

[57] Zhang D., Shi D., Wang F., Qian D., Zhou Y., Fu J., Chen M., Qiu D., Jiang S. (2023). Electromagnetic shocking induced fatigue improvement via tailoring the *α*-grain boundary in metastable *β* titanium alloy bolts. J. Alloys Compd..

[58] Joy R., Wu S.h., Tariq U., Mahmood M.A. Effect of Inter-Layer Dwell Time on Residual Stresses in Directed Energy Deposition of High Strength Steel Alloy. Proceedings of the Solid Freeform Fabrication Symposium—An Additive Manufacturing Conference.

[59] Fu Z., Yang B., Shan M., Li T., Zhu Z., Ma C., Zhang X., Gou G., Wang Z., Gao W. (2020). Hydrogen embrittlement behavior of SUS301L-MT stainless steel laser-arc hybrid welded joint localized zones. Corros. Sci..

[60] Fang J., Ma G., Tian H., Li S., Huang H., Liu Y., Jiang Y., Liu B. (2021). Transformation-induced strain of a low transformation temperature alloy with high hardness during laser metal deposition. J. Manuf. Processes.

[61] Goldak J., Chakravarti A., Bibby M. (1984). A new finite element model for welding heat sources. Metall. Trans. B.

[62] Gouge M., Michaleris P., Denlinger E., Irwin J. (2018). The finite element method for the thermo-mechanical modeling of additive manufacturing processes. Thermo-Mechanical Modeling of Additive Manufacturing.

[63] Jiang Y., Fang J., Ma G., Tian H., Zhang D., Cao Y. (2021). Microstructure and properties of an as-deposited and post treated high strength carbide-free bainite steel fabricated via laser powder deposition. Mater. Sci. Eng. A.

[64] Heigel J.C., Michaleris P., Palmer T.A. (2016). Measurement of forced surface convection in directed energy deposition additive manufacturing. Proc. Inst. Mech. Eng. B Part J. Eng. Manuf..

[65] Virag Z., Živić M., Krizmanić S. (2011). Cooling of a sphere by natural convection–The applicability of the lumped capacitance method. Int. J. Heat Mass Transf..

[66] Gouge M.F., Heigel J.C., Michaleris P., Palmer T.A. (2015). Modeling forced convection in the thermal simulation of laser cladding processes. Int. J. Adv. Manuf. Technol..

[67] Dunbar A.J., Denlinger E.R., Gouge M.F., Michaleris P. (2016). Experimental validation of finite element modeling for laser powder bed fusion deformation. Addit. Manuf..

[68] Lindgren L.E. (2001). Finite element modeling and simulation of welding part 1: Increased complexity. J. Therm. Stress..

[69] Michaleris P. (2014). Modeling metal deposition in heat transfer analyses of additive manufacturing processes. Finite Elem. Anal. Des..

[70] Ning J., Praniewicz M., Wang W., Dobbs J.R., Liang S.Y. (2020). Analytical modeling of part distortion in metal additive manufacturing. Int. J. Adv. Manuf. Technol..

[71] Sadik S., Yavari A. (2017). Geometric nonlinear thermoelasticity and the time evolution of thermal stresses. Math. Mech. Solids.

[72] Khan K., Mohan L.S., De A., DebRoy T. (2023). Rapid calculation of part scale residual stresses in powder bed additive manufacturing. Sci. Technol. Weld. Join..

[73] Li C., Liu J., Guo Y. (2016). Prediction of residual stress and part distortion in selective laser melting. Procedia CIRP.

[74] Kim T., Ha K., Cho Y.R., Jeon J.B., Lee W. (2019). Analysis of residual stress evolution during powder bed fusionprocess of AISI 316L stainless steel with experiment and numerical modeling. Int. J. Adv. Manuf. Technol..

[75] Smith W.L., Roehling J.D., Strantza M., Ganeriwala R.K., Ashby A.S., Vrancken B., Clausen B., Guss G.M., Brown D.W., McKeown J.T. (2021). Residual stress analysis of in situ surface layer heating effects on laser powder bed fusion of 316L stainless steel. Addit. Manuf..

[76] Cheng B., Shrestha S., Chou K. (2016). Stress and deformation evaluations of scanning strategy effect in selective laser melting. Addit. Manuf..

[77] Li Y., Zhou K., Tan P., Tor S.B., Chua C.K., Leong K.F. (2018). Modeling temperature and residual stress fields in selective laser melting. Int. J. Mech. Sci..

[78] Dai K., Shaw L. (2001). Thermal and stress modeling of multi-material laser processing. Acta Mater..

[79] Gu D., He B. (2016). Finite element simulation and experimental investigation of residual stresses in selective laser melted Ti–Ni shape memory alloy. Comput. Mater. Sci..

[80] Marques B.M., Andrade C.M., Neto D.M., Oliveira M.C., Alves J.L., Menezes L.F. (2020). Numerical analysis of residual stresses in parts produced by selective laser melting process. Procedia Manuf..

[81] Fergani O., Berto F., Welo T., Liang S. (2017). Analytical modelling of residual stress in additive manufacturing. Fatigue Fract. Eng. Mater. Struct..

[82] Mirkoohi E., Li D., Garmestani H., Liang S.Y. (2020). Analytical modeling of residual stress in laser powder bed fusion considering volume conservation in plastic deformation. Modelling.

[83] Bailey N.S., Katinas C., Shin Y.C. (2017). Laser direct deposition of AISI H13 tool steel powder with numerical modeling of solid phase transformation, hardness, and residual stresses. J. Mater. Process. Technol..

[84] Lu X., Chiumenti M., Cervera M., Li J., Lin X., Ma L., Zhang G., Liang E. (2021). Substrate design to minimize residual stresses in Directed Energy Deposition AM processes. Mater. Des..

[85] Jing H., Ge P., Zhang Z., Chen J.Q., Liu Z.M., Liu W.W. (2022). Numerical studies of the effects of the substrate structure on the residual stress in laser directed energy additive manufacturing of thin-walled products. Metals.

[86] Megahed M., Mindt H.W., N’Dri N., Duan H., Desmaison O. (2016). Metal additive-manufacturing process and residual stress modeling. Integr. Mater. Manuf. Innov..

[87] Tariq U., Joy R., Wu S.H., Mahmood M.A., Malik A.W., Liou F. (2023). A state-of-the-art digital factory integrating digital twin for laser additive and subtractive manufacturing processes. Rapid Prototyp. J..

[88] Mahmood M.A., Tariq U. (2023). A novel framework using FEM and machine learning models with experimental verification for Inconel-718 rapid part qualification by laser powder bed fusion. Int. J. Adv. Manuf. Technol..

[89] Wu S.h., Joy R., Tariq U., Mahmood M.A. Role of In-situ Monitoring Technique for Digital Twin Development using Direct Energy Deposition: Melt Pool Dynamics and Thermal Distribution. Proceedings of the Solid Freeform Fabrication Symposium—An Additive Manufacturing Conference.

[90] Zaid M.M., Xu G., Amoah N. (2023). Accuracy of low-cost particulate matter sensor in measuring coal mine dust-a wind tunnel evaluation. Underground Ventilation.

[91] Zhang J., Yu M., Li Z., Liu Y., Zhang Q., Jiang R., Sun S. (2021). The effect of laser energy density on the microstructure, residual stress and phase composition of H13 steel treated by laser surface melting. J. Alloys Compd..

[92] Balbaa M., Nasr M.N., Elgamal H. (2017). A Sensitivity Analysis on the Effect of Laser Power on Residual Stresses When Laser-assisted Machining AISI 4340. Procedia CIRP.

[93] Ali H., Ghadbeigi H., Mumtaz K. (2018). Effect of scanning strategies on residual stress and mechanical properties of Selective Laser Melted Ti6Al4V. Mater. Sci. Eng. A.

[94] Bian P., Shi J., Liu Y., Xie Y. (2020). Influence of laser power and scanning strategy on residual stress distribution in additively manufactured 316L steel. Opt. Laser Technol..

[95] Mugwagwa L., Yadroitsev I., Matope S. (2019). Effect of process parameters on residual stresses, distortions, and porosity in selective laser melting of maraging steel 300. Metals.

[96] Ali H., Ghadbeigi H., Mumtaz K. (2018). Processing Parameter Effects on Residual Stress and Mechanical Properties of Selective Laser Melted Ti_6_Al_4_V. J. Mater. Eng. Perform..

[97] Wang Z., Denlinger E., Michaleris P., Stoica A.D., Ma D., Beese A.M. (2017). Residual stress mapping in Inconel 625 fabricated through additive manufacturing: Method for neutron diffraction measurements to validate thermomechanical model predictions. Mater. Des..

[98] Pandey A., Gaur V. (2023). Effect of dwell time on fatigue properties of wire-arc additively manufactured IN718 alloy. Int. J. Fatigue.

[99] Ivanov S., Vildanov A., Golovin P., Artinov A., Karpov I. (2019). Effect of inter-layer dwell time on distortion and residual stresses of laser metal deposited wall. Key Eng. Mater..

[100] Mirkoohi E., Dobbs J.R., Liang S.Y. (2020). Analytical modeling of residual stress in direct metal deposition considering scan strategy. Int. J. Adv. Manuf. Technol..

[101] Robinson J., Ashton I., Fox P., Jones E., Sutcliffe C. (2018). Determination of the effect of scan strategy on residual stress in laser powder bed fusion additive manufacturing. Addit. Manuf..

[102] Strantza M., Ganeriwala R.K., Clausen B., Phan T.Q., Levine L.E., Pagan D.C., Ruff J.P., King W.E., Johnson N.S., Martinez R.M. (2021). Effect of the scanning strategy on the formation of residual stresses in additively manufactured Ti-_6_Al-_4_V. Addit. Manuf..

[103] Promoppatum P., Yao S.C. (2020). Influence of scanning length and energy input on residual stress reduction in metal additive manufacturing: Numerical and experimental studies. J. Manuf. Processes.

[104] Nadammal N., Mishurova T., Fritsch T., Serrano-Munoz I., Kromm A., Haberland C., Portella P.D., Bruno G. (2021). Critical role of scan strategies on the development of microstructure, texture, and residual stresses during laser powder bed fusion additive manufacturing. Addit. Manuf..

[105] He B., Bi C., Li X., Wang W., Yang G. (2023). Residual stresses and deformations of laser additive manufactured metal parts: A review. Int. J. Mater. Form..

[106] Joy R., Wu S.h., Tariq U., Mahmood M.A. State-of-the-art Cyber-enabled Physical and Digital Systems Deployed in Distributed Digital Factory Using Additive and Subtractive Manufacturing Systems: Open, Scalable, and Secure Framework. Proceedings of the Solid Freeform Fabrication Symposium—An Additive Manufacturing Conference.

[107] Satpathy M.P., Mishra S.B., Sahoo S.K. (2018). Ultrasonic spot welding of aluminum-copper dissimilar metals: A study on joint strength by experimentation and machine learning techniques. J. Manuf. Processes.

[108] Gong L., Huang S., Yang X., Zhang Z., Liu K. (2017). Effect of welding residual stress on properties of Cr-Mo steel. Bol. Tec. Bull..

[109] Olabi A.G., Lostado R., Benyounis K.Y. (2014). Review of Microstructures, Mechanical Properties, and Residual Stresses of Ferritic and Martensitic Stainless-Steel Welded Joints.

[110] Das D., Das A.K., Pratihar D., Roy G. (2021). Prediction of residual stress in electron beam welding of stainless steel from process parameters and natural frequency of vibrations using machine-learning algorithms. Proc. Inst. Mech. Eng. Part C J. Mech. Eng. Sci..

[111] Edwin Raja Dhas J., Kumanan S. (2016). Evolutionary fuzzy SVR modeling of weld residual stress. Appl. Soft Comput. J..

[112] Mathew J., Griffin J., Alamaniotis M., Kanarachos S., Fitzpatrick M.E. (2018). Prediction of welding residual stresses using machine learning: Comparison between neural networks and neuro-fuzzy systems. Appl. Soft Comput. J..

[113] Wacker C., Köhler M., David M., Aschersleben F., Gabriel F., Hensel J., Dilger K., Dröder K. (2021). Geometry and distortion prediction of multiple layers for wire arc additive manufacturing with artificial neural networks. Appl. Sci..

[114] Wu Q., Mukherjee T., De A., DebRoy T. (2020). Residual stresses in wire-arc additive manufacturing – Hierarchy of influential variables. Addit. Manuf..

[115] Zhou Z., Shen H., Liu B., Du W., Jin J., Lin J. (2022). Residual thermal stress prediction for continuous tool-paths in wire-arc additive manufacturing: A three-level data-driven method. Virtual Phys. Prototyp..

[116] Hajializadeh F., Ince A. (2021). Integration of artificial neural network with finite element analysis for residual stress prediction of direct metal deposition process. Mater. Today Commun..

[117] Zhu Z., Ferreira K., Anwer N., Mathieu L., Guo K., Qiao L. (2020). Convolutional Neural Network for geometric deviation prediction in Additive Manufacturing. Procedia CIRP.

[118] Ralph B.J., Hartl K., Sorger M., Schwarz-Gsaxner A., Stockinger M. (2021). Machine learning driven prediction of residual stresses for the shot peening process using a finite element based grey-box model approach. J. Manuf. Mater. Process..

[119] Peter N., Pitts Z., Thompson S., Saharan A. (2020). Benchmarking build simulation software for laser powder bed fusion of metals. Addit. Manuf..

[120] Thakur S., Talla G., Verma P. (2021). Residual stress, distortion, and porosity analysis of LED heat sink printed by SLM process using machine learniNg. Eng. Res. Express.

[121] Khadilkar A., Wang J., Rai R. (2019). Deep learning–based stress prediction for bottom-up SLA 3D printing process. Int. J. Adv. Manuf. Technol..

[122] Dong G., Wong J.C., Lestandi L., Mikula J., Vastola G., Jhon M.H., Dao M.H., Kizhakkinan U., Ford C.S., Rosen D.W. (2022). A part-scale, feature-based surrogate model for residual stresses in the laser powder bed fusion process. J. Mater. Process. Technol..

[123] Francis J., Bian L. (2019). Deep Learning for Distortion Prediction in Laser-Based Additive Manufacturing using Big Data. Manuf. Lett..

[124] Ansari M.A., Crampton A., Parkinson S. (2022). A Layer-Wise Surface Deformation Defect Detection by Convolutional Neural Networks in Laser Powder-Bed Fusion Images. Materials.

[125] Chen Q. (2021). Multiscale Process Modeling of Residual Deformation and Defect Formation for Laser Powder Bed Fusion Additive Manufacturing. Ph.D. Thesis.

[126] Koeppe A., Hernandez Padilla C.A., Voshage M., Schleifenbaum J.H., Markert B. (2018). Efficient numerical modeling of 3D-printed lattice-cell structures using neural networks. Manuf. Lett..

[127] Gong X., Zeng D., Groeneveld-Meijer W., Manogharan G. (2022). Additive manufacturing: A machine learning model of process-structure-property linkages for machining behavior of Ti-6Al-4V. Mater. Sci. Addit. Manuf..

[128] Liao S., Xue T., Jeong J., Webster S., Ehmann K., Cao J. (2023). Hybrid thermal modeling of additive manufacturing processes using physics-informed neural networks for temperature prediction and parameter identification. Comput. Mech..

[129] Salvati E., Tognan A., Laurenti L., Pelegatti M., De Bona F. (2022). A defect-based physics-informed machine learning framework for fatigue finite life prediction in additive manufacturing. Mater. Des..

[130] Almeida H.A., Vasco J.C., Marto A., Capela C., Freitas D., Craveiro F., Bártolo H., Coelho L., Correia M., Vieira M. (2020). Progress in Digital and Physical Manufacturing.

